# An eye movement pre-training fosters the comprehension of processes and functions in technical systems

**DOI:** 10.3389/fpsyg.2015.00598

**Published:** 2015-05-12

**Authors:** Irene T. Skuballa, Caroline Fortunski, Alexander Renkl

**Affiliations:** ^1^Department of Applied Cognitive Psychology and Media Psychology, University of TübingenTübingen, Germany; ^2^Educational and Developmental Psychology, University of FreiburgFreiburg, Germany

**Keywords:** eye movement, pre-training, top-down process, stress, learning

## Abstract

The main research goal of the present study was to investigate in how far pre-training eye movements can facilitate knowledge acquisition in multimedia (pre-training principle). We combined considerations from research on eye movement modeling and pre-training to design and test a non-verbal eye movement-based pre-training. Participants in the experimental condition watched an animated circle moving in close spatial resemblance to a static visualization of a solar plant accompanied by a narration in a subsequently presented learning environment. This training was expected to foster top-down processes as reflected in gaze behavior during the learning process and enhance knowledge acquisition. We compared two groups (*N* = 45): participants in the experimental condition received pre-training in a first step and processed the learning material in a second step, whereas the control group underwent the second step without any pre-training. The pre-training group outperformed the control group in their learning outcomes, particularly in knowledge about processes and functions of the solar plant. However, the superior learning outcomes in the pre-training group could not be explained by eye-movement patterns. Furthermore, the pre-training moderated the relationship between experienced stress and learning outcomes. In the control group, high stress levels hindered learning, which was not found for the pre-training group. On a delayed posttest participants were requested to draw a picture of the learning content. Despite a non-significant effect of training on the quality of drawings, the pre-training showed associations between learning outcomes at the first testing time and process-related aspects in the quality of their drawings. Overall, non-verbal pre-training is a successful instructional intervention to promote learning processes in novices although these processes did not directly reflect in learners' eye movement behavior during learning.

## Introduction

The present study investigates a non-verbal eye movement pre-training on learning. This instructional intervention was designed to foster the comprehension of processes and functions in a static representation of a technical system. Research on the pre-training principle in multimedia demonstrates that prior knowledge provokes top-down processes and can enhance understanding of unfamiliar materials (Mayer, 2009). Feedforward trainings and modeling of eye movements were shown successful in guiding learners' visual attention and in enhancing comprehension (Nalanagula et al., 2006; Jarodzka et al., 2013). Based on these findings, we introduce a pre-training which provides prior knowledge about motions of flow in the to-be-learned technical system by guiding learners' eye movements in a content-free environment without verbalizing any further information. The eye movement pre-training is characterized by dynamic events through guided eye movements that should be originally and “naturally” accomplished when actively processing the displayed technical system by mental simulation.

The rationale behind our research was to manipulate top-down processes via prior knowledge about dynamic processes (Kriz and Hegarty, 2007; Lowe and Boucheix, 2008). We tested whether the pre-training would have a transfer effect on the subsequently presented technical system and, as a consequence, lead to better learning outcomes. In addition, we tested the effects of pre-training on learners' eye movement behavior, cognitive load, and experienced stress levels during the learning phase.

## Importance of prior knowledge

The perception of an external stimulus presented on a screen marks the starting point of multimedia learning (Hegarty, 2005; Mayer, 2009). This learning process is guided actively through enhancing attention allocation processes. Only information (i.e., visual and auditory) that is actively processed can be encoded and incorporated into an internal representation of the subject matter. During the course of learning different pieces of information are connected with learners' prior knowledge and integrated into an internal mental representation.

The framework proposed by Kriz and Hegarty (2007) distinguishes two perspectives on the comprehension of multimedia: a top-down and a bottom-up perspective. The bottom-up perspective interprets learning as a result of the quality of the displayed representation. Bottom-up processes are automatic and driven by properties inherent in stimuli attracting the learner's attention. This means that learners do not spontaneously attend to the most relevant information but rather to most prominent, salient, or distractive stimuli (Lowe and Boucheix, 2008). These processes can be manipulated by instructional design. Design principles such as arrows, spotlights, or instructions aim to guide learners' visual attention allocation to predefined relevant information areas (Mautone and Mayer, 2007; Plass et al., 2009). The addition of non-content information, however, is controversial. Cues may prevent a holistic view and do not necessarily lead to better learning outcomes (De Koning et al., 2010). In line with these reservations, Kriz and Hegarty (2007) found that manipulating bottom-up processes by adding interactivity or arrows to an animation did not successfully enhance learning performance.

Top-down processes, on the other hand, are regulated by the learner's prior knowledge (Kriz and Hegarty, 2007). Perception and encoding of new learning contents are determined by the accuracy and quantity of preexisting knowledge. Equipping learners with knowledge can guide their attention and thus lead to top-down processing: participants with higher levels of domain knowledge were better in constructing accurate mental models and in revising previous knowledge deficits, that means, identifying and closing knowledge gaps (Kriz and Hegarty, 2007). Top-down processing is reflected in the pre-training principle.

## The pre-training principle

Presenting content-specific information prior to a main learning phase aims to promote top-down processes. It prevents learners from being distracted by salient but less relevant information when learning, relieves cognitive load and thus facilitates learning performance, especially in high-element interactivity environments when the visual and auditory channels are already working to capacity (Mayer and Moreno, 2003; Sweller et al., 2011). This strategy is known as pre-training and refers to delivery of preceding information on components or structures of the learning content to foster the generation of initial basic knowledge (Mayer et al., 2002). The benefits of pre-training are interpreted in terms of a two-stage theory of mental model construction. According to this model, learners should construct a mental representation of a component model in a first stage followed by a model of the entire system in a second stage (Mayer et al., 2002). The first stage aims at teaching learners about the isolated components of a system and their changes on a local level; the second stage aims at teaching learners the cause-and-effect relationships between the previously learned components on a global level (Mayer et al., 2002).

The pre-training principle was confirmed in several experiments on learning about technical systems. Positive effects of a pre-training treatment were found in paper-pencil, computer-based, and hands-on learning environments as well as with static and animated learning materials (Mayer et al., 2002; Pollock et al., 2002). In the aforementioned experiments pre-training equipped learners with declarative knowledge about the structures or components of the respective systems but not with knowledge about the global dynamic processes. The learning environments demonstrated processes and functions, whereas the components were learned beforehand in pre-training. It remains unclear whether pre-training is solely effective in providing prior knowledge on components or whether other domain-specific and relevant features such as the processes of the system can also be pre-trained. As many technical systems explain dynamic processes and flow directions, it is worth considering whether pre-training can also provide knowledge on motion events.

When a representation is presented in a static manner and, thus, visual dynamics are absent, learners must mentally animate the motions to infer processes and mechanics (Hegarty, 1992). In an attempt to foster the mental simulation of motions in a technical system, we developed a gaze based pre-training. We examined whether a non-verbal pre-training on eye movements could positively influence the construction of a mental model from a static representation in order to foster comprehension of the dynamics in a technical system.

## Learning by seeing through an expert's eyes

Type of task and level of expertise can affect gaze behavior. Experts and novices not only systematically differ in their performance as a result of their actions but also in the eye movement patterns that lead to their outcomes (e.g., Charness et al., 2001). For instance, a meta-analysis on visual expertise by Gegenfurtner et al. (2011) showed that experts processed information faster and neglected task-irrelevant information for the benefit of task-relevant information. Experts' information processing was manifested in shorter fixation durations and a selective attention on task-relevant information. These results lead to the question whether expertise can be acquired faster when novices observe the eye movements of an expert (Gegenfurtner et al., 2011).

The presentation of experts' gaze to novices can facilitate search performance. In terms of feedforward training, Nalanagula et al. (2006) demonstrated how eye movements can be successfully implemented to pre-train novices in a visual inspection task. The tested feedforward training applied eye movement data as prior information to promote search performance in novices. Feedforward training refers to the provision of task-dependent knowledge before a person performs a task. Here, the task was to detect defects in visually presented circuit boards. For the feedforward training the authors, first, recorded the eye movements of an expert performing the search task and, then, superimposed the expert's scanpath on the visual stimulus, namely the circuit board. Before inspecting circuit boards and detecting defects themselves, participants received one of three trainings. One group saw a static of a circuit board with a static and complete scanpath representing eye movement behavior (static condition), another group watched a circle representing the fixation of an eye superimposed on the same material (dynamic condition), and a third group watched a dynamic scanpath with a circle which developed over the time of inspection (hybrid condition). Afterwards participants had to search for defects in unfamiliar circuit boards. Overall, a dynamic and hybrid feedforward training had beneficial transfer effects on the new tasks. Participants in these conditions outperformed the static group and a control group without training. The authors conclude that the eye movements in the training might have offered insight into the models performance processes making easier to the novices to understand the processes of the inspection task. As the participants' eye movement data were not analyzed, it remained unanswered whether the beneficial effects of the feedforward training were due to a mediation of the participants' gaze behavior. Similarly, Litchfield and colleagues showed in a series of experiments that novices who observed the eye movements of an expert who was searching for pulmonary nodules in chest x-rays identified more nodules correctly when they had to search for nodules themselves (Litchfield et al., 2010). More specifically it was shown that it was not the model's expertise but the task-specificity of eye movements in the pre-training that fostered search performance. Taken together, watching a model's eye movements functions as a scaffold. The model's eye movements were marked by specific eye movement patterns, however, no information was provided on transfer of the training on the gaze behavior performed by the participants who observed the models. Novices might have profited from the prior information because they reenacted the experts' gaze. Alternatively, they might have profited from the training without having to perform the same eye movement patterns.

Findings on eye movement modeling examples (EMMEs) show similarly promising results plus a slight transfer effect on gaze behavior. EMMEs can be created by recording the experts' eye movements when performing a visual task and replaying these recordings superimposed on the learning content (Jarodzka et al., 2012). Such EMMEs can be considered as worked-out examples demonstrating where and when to look at specific regions of the learning content. EMMEs visualize an expert model's eye movements on a stimulus providing the basis for information processes and verbal explanations of the model. It is suggested that observing experts how they are selecting and organizing visual information could elicit the same processes in a novice. Seppänen and Gegenfurtner (2012) asked participants to interpret a computer tomography scan. Participants who watched a video replaying an expert's eye movements augmented by verbal interpretations of such a scan improved in diagnosing, fixated more task-relevant information and less task-redundant information. Modeling eye movements by spotlights guided attention more successfully and helped learners to identify relevant information at an earlier stage as well as fixate task-relevant information for a longer time (Jarodzka et al., 2010a, 2012). Other studies demonstrated that, in addition to the superior learning outcomes, this type of eye movement training can provoke more similar, that is coherent, eye movement scanpaths within a training condition (Jarodzka et al., 2013).

Cognitive guidance via gaze behavior can be successful even without verbalized directions (Grant and Spivey, 2003). In an experiment on problem solving, the eye movements of successful problem-solvers were used to develop cues for participants who were unfamiliar to the problem. Short flashes were implemented as cues to attract attention and trigger useful and beneficial eye movements for solving the task. Participants who were exposed to the gaze attracting cues outperformed participants who learned with noncritical cues or no cues. Based on these results, Thomas and Lleras (2007) made an attempt to facilitate successful problem solving by making their participants “move their eyes in a pattern that embodied the problem's solution” (p. 664). Comparing several conditions, the authors not only corroborated the results by Grant and Spivey (2003), but also found a relationship between eye movements and spatial cognition: the closer the resemblance between the spatiality of real eye movements and cues, the better the solution rate of learners. Learners were not aware of the connection between the cues and eye movements.

Even though these studies do not provide instructional information on the components of the learning contents, the results can be explained within the two-stage framework of mental model construction (Mayer et al., 2002). Here, we can generalize the model as follows: In a first stage, learners acquire prior knowledge which enables them to build an initial and possibly incomplete mental model which is not restricted to components only. In a second stage, further incoming information can be encoded more easily and integrated with the schemata constructed during the first stage.

## Knowledge about technical systems

Dynamics and movements are prominent and specific attributes of physical, technical, mechanical, and even business systems (Clark et al., 2006). To understand how a technical system works learners must understand the processes and motions underlying the technical device which, in turn, can determine its functions.

Comprehension of technical systems comprises different types of knowledge (Hegarty, 2005). First, learners must acquaint themselves with the components of a technical system. Based on the configuration of the system's parts, learners can construct a static model of the technical system (Hegarty, 2005). Next, learners must acquire knowledge of the movements and causal interdependencies within the technical system. Based on knowledge about the behaviors and processes of a system, learners can construct a dynamic mental model of the system. The functions of a technical system describe its purpose and how the structures and processes of the system operate in order to make it functional in attending to its purpose. Last, by combining knowledge of its structures, processes, and functions, learners can construct a full internal representation of a technical device (Hegarty, 2005). In line with these observations, Kalyuga and Hanham (2011) argue for a functions-processes-structures framework when describing technical systems to novice learners. Whereas structures are visible information pieces which are easy to access and process, processes and functions must often be inferred and, therefore, represent deeper understanding (Hmelo-Silver and Pfeffer, 2004). Due to its relevance and challenges for mental animation we decided to use a static visual representation of a technical system, a solar plant, to test the effectiveness of our pre-training. In addition, we applied the functions-processes-structures framework to capture comprehension.

## The present study

Against the backdrop of these considerations we developed a pre-training that tracked the dynamics in a technical system explained in a narration. The pre-training required participants to follow a black circle which was moving in spatial correspondence to the contents of the learning environment. However, the background was gray and contents were not revealed during the pre-training phase. After the pre-training, participants listened to a narration explaining the structures, processes, and functions of a solar plant while they were watching a static representation of the system. We compared two conditions: the experimental condition watched the pre-training in a first step and the learning environment on a solar plant in a second step; the control condition watched the learning environment on a solar plant without any preceding information. The pre-training was presented in a dynamic way where the circle cue moved around; the learning environment was presented in a static visualization. We addressed the following expectations:

### Learning outcomes

According to the functions-processes-structures framework (Hmelo-Silver and Pfeffer, 2004; Hegarty, 2005; Kalyuga and Hanham, 2011), we distinguished between structures, processes, and functions when assessing knowledge. Adding extra interventions such as pre-training should result in better learning outcomes. We therefore expected the pre-training group to outperform the no-training group (i.e., control group) on overall learning outcomes. Next, we examined the different knowledge types for technical systems (i.e., knowledge about structures, processes, and functions). As the pre-training provided information on motions and causal relationships we expected the pre-training group to achieve better learning outcomes on processes. In addition, we analyzed knowledge about functions and structures separately.

### Self-report measures on experiences during learning

It is predicted that a pre-training intervention can unfold its facilitating effects on comprehension processes by reducing adverse experiences such as cognitive load and mental effort during learning (Paas and van Merriënboer, 1993; Sweller et al., 2011). Cognitive load and mental effort can be measured by asking learners how difficult it was to comprehend or to study the instructional material. Complementary, experienced stress can have also detrimental effects on learning outcomes. Experienced stress during learning can impair learning in terms of memorizing, retention, and recall: recent research demonstrates that stress has a negative influence on the quantity and quality of memory formation in learning situations (Schwabe et al., 2010). More specifically, it was found that the different effects of stress on learning are time-dependent and can affect encoding, consolidation, retrieval, and reconsolidation processes (Schwabe et al., 2012). Self-report measures of stress are time-saving and valid (Schwabe and Wolf, 2010). We expected that pre-training would result in lower levels of cognitive load, mental effort, and experienced stress.

### Eye movement behavior

Finally, we were interested in how far a pre-training based on eye movements could impact eye movements performed during the learning phase. As part of our manipulation check, we first examined whether and for how long participants in the pre-training group followed the circle cue from the pre-training stage that primed the processes in the subsequent learning environment.

For all analyses of eye movements we defined semantic areas of interest (AOIs) in the learning environment. AOIs were bound to visual key information that was crucial for comprehension and understanding of how the technical device was composed and of how it worked. Because the pre-training should enhance the awareness for content-relevant information, we expected the pre-training group to show longer dwell times on task relevant AOIs in the learning environment. Next, we examined how the pre-training would reflect on the saccades performed during the inspection of the learning environment. We expected the pre-training group to demonstrate more saccades in the direction of the fluids of the to-be-learned technical system as it was described by the narration. Finally, since the eye movement pre-training trained participants' eye movements to perform a specific order of movements as predetermined by the learning environment, we expected eye movements in the pre-training group to be more homogeneous in terms of similarity of strings analyzed by the Levenshtein distance.

### Delayed posttest

Some benefits of multimedia learning principles can decline over time (Schweppe and Rummer, 2012). Therefore, we asked our participants to take part in a follow-up posttest 1 week later. Participants were required to create a picture of the learning content by implementing arrows to represent the direction of motion in the system. The quality of the drawings was scored and compared in an explorative manner. We had no specific expectations in this respect.

## Method

### Sample and design

Participants were 45 psychology students from the University of Freiburg who received course credit points for participation. They were randomly assigned either to an experimental group with eye movement pre-training prior to the learning phase or a control group without such pre-training. Neither participants nor experimenters were aware of the purpose of the study. The procedure was highly standardized by reducing personal contact between participant and experimenter to a minimum. Instructions were provided in written form on paper or screen. One participant in the pre-training group disclosed the purpose of the experiment and was thus excluded from the analysis, leaving 22 participants in each condition for our analysis (36 female; *M*_age_ = 22.67, *SD* = 4.81). Participants were tested in individual sessions of approximately 60 min. This experiment was conducted in accordance with the German Psychological Society (DGPs) ethical guidelines (2004, CIII) and the APA ethical standards. Participation was voluntary and confidential. Data collection was anonymized by participant codes. Participants provided verbal informed consent and could withdraw at any time without consequences. Contact information from the research team was provided to give participants the opportunity to withdraw their data or ask for further information following the experiment. After data collection was finished, participants were informed about the experiment's purpose and previous results in a lecture on educational psychology.

### Learning material

**Figure 1:**
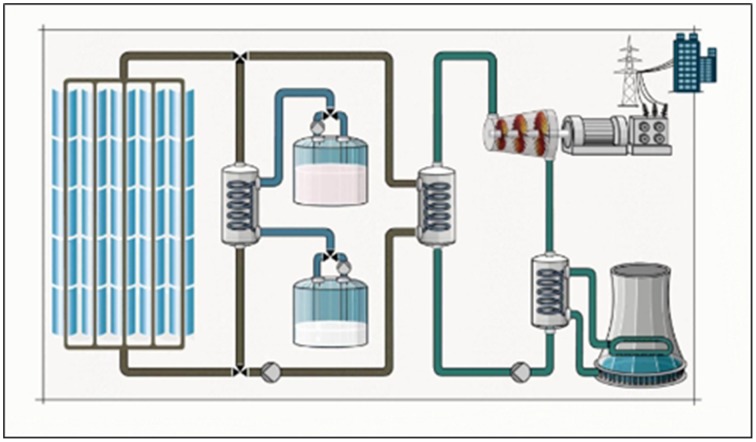
**Screenshot of the learning environment: solar plant (adapted from http://www.solarmillennium-invest.de/cms/upload/Flash/andasol_blue.swf)**.

The learning material consisted of a static and plane picture of a solar power plant illustrating the conversion of solar radiation into electricity (Figure 1). The representation was accompanied by an audio narration presented via headphones. An excerpt from the narration is provided in the Appendix.

The learning environment showed three operational and temporarily overlapping cycles. First, the topic and its relevance were introduced, then, the cycles of the system and their interdependencies were detailed. The components (i.e., structures) were labeled—facilitating orientation—and referred to the individual parts of the technical device. The pipes of the system are filled with different fluids which move in various directions through the course of energy conversion. These motions, however, were not displayed in the visual representation, but described by the narration. The flow of the fluids, as explained in the narration, was taken as an anchor for the pre-training. This material was chosen for two reasons. First, to understand the solar plant learners must mentally simulate dynamic processes. Second, it offers dense information and causal relationships within the material so that full comprehension might be challenging without any additional support in form of cueing or, in this case, pre-training.

### Pre-training

**Figure 2:**
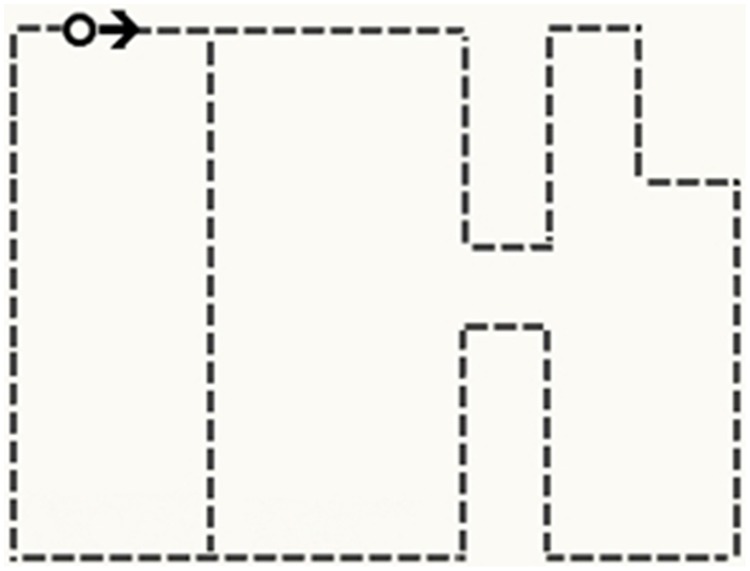
**Schematic illustration of the eye movement pre-training**. The black circle represents the animated stimulus for the pre-training, the black arrow represents the direction of the animated stimulus, and the dashed lines represent its movements. Note that neither arrow nor dashed lines were visible to the participants.

The eye movement pre-training in the experimental condition contained an animated single black circle (0.3 cm in diameter) moving smoothly and analogically to the pipes of the technical device that was presented in the subsequent learning phase (see Video 1 in Supplement Data). Note, however, that during the whole pre-training the background of the screen did not display the actual learning environment (see Figure 2 and Video 1). So, contrary to the feedforward training by Nalanagula et al. (2006), the task was not visible.

The direction and order of the circle's movements were congruent to the narration that accompanied the learning material. The black circle in the pre-training was identical to the stimulus used during the calibration process of the eye tracking device. Participants in the pre-training group were instructed to follow the circle as they were previously doing for the calibration process. The close spatial resemblance between pre-training and the upcoming learning environment, however, was not revealed in the instructions. The pre-training lasted for about 2 min and simulated the movements of all three causal cycles depicted by the solar plant. The pre-training aimed at promoting attention to the processes of the technical system and thereby at fostering the understanding of the causal relationships between the components.

### Prior knowledge

When testing new instructional design formats, it is important to take learners' prior knowledge into account because it can moderate the beneficial effects of instructional design (expertise reversal effect, e.g., Kalyuga, 2007). We developed a test with open questions on domain-specific contents related to the learning material to assess prior knowledge. Overall, prior knowledge as assessed by the pretest was very low, *M* = 5.83 (percentage score), *SD* = 3.60, indicating that learners had hardly any prior knowledge about the tested learning contents. Since the learning material contained technical concepts of heat transfer and movements, we asked the participants to report their last school grade in physics (1 = *very good*; 6 = *fail*) as an indicator of general domain knowledge. Prior knowledge and last grade in physics were assessed as potential predictors and covariates for the learning outcomes.

### Learning outcomes

To assess learning outcomes, we developed a test based on the functions-processes-structures framework by Kalyuga and Hanham (2011). The test consisted of open questions on the domain-specific content focusing on structures, processes, and functions. Structures are the components a technical device contains, processes are operations within a device, and functions refer to the purpose the components serve. The structures corresponded to the labeled components directly presented in the learning material (e.g., Please, name the components of a solar plant.) whereas the processes (e.g., Please, describe the processes in the water-steam cycle.) and functions (e.g., Please, describe how a turbine works.) could be deduced from the narration in combination with the visual stimulus. Structures, processes, and functions were assessed by three questions each. The answer to each question consisted of several aspects or items, respectively. All answers were scored by a rater unaware of the participant's condition. A subset of 25% was scored by a second rater and revealed high interrater reliability (ICC using mixed model, absolute type): 15 items on structures, *ICC* = 0.989, 15 items on processes: *ICC* = 0.959, 10 items on functions: *ICC* = 0.958. All disagreements were resolved by consensus.

### Load, mental effort, and stress measurement

Self-report measures referred to the experience during the learning phase and were assessed with three items on a 9-point-Likert scale. Participants were asked to indicate how difficult it was to comprehend the presented learning content (load measure: 1 = *not at all difficult*; 9 = *very difficult*), how much mental effort they invested to comprehend the learning environment (mental effort: 1 = *not at all*; 9 = *very much*), and how stressed they felt during the learning process (stress: 1 = *not at all stressed*; 9 = *very stressed*).

### Apparatus

Gaze data were recorded by a SensoMotoric Instruments Remote Eye-tracking Device and iView X 2.7 (120 Hz, angular error < 0.5). The stimulus was presented via ExperimentCenter 3.0 (22″ monitor, display resolution of 1680 × 1050, set 60–80 cm in front of the participant). For the export of the gaze data we used BeGaze 3.0 software (www.smivision.com).

### Procedure

First, participants answered a questionnaire on demographics and worked on a test on prior knowledge. After calibration, participants were randomly assigned to either a condition with pre-training or a condition without pre-training. While the pre-training group was instructed to follow a black circle on a content-free screen prior to the learning environment (Figure 2), the control group continued immediately with the learning environment. After the learning phase, participants were asked to report how difficult it was to comprehend the presented learning contents to assess cognitive load, *M* = 3.89, *SD* = 2.10, how much mental effort they have invested, *M* = 4.91, *SD* = 2.03, and how stressed they felt during the learning unit, *M* = 3.82, *SD* = 2.18. Then, learning outcomes were assessed by a domain-specific posttest. There was no time limit for knowledge assessment, neither for the prior knowledge test nor for the learning outcomes posttest. Participants in the pre-training group were requested to reproduce the direction and events of the pre-training on a sheet of paper. In the final steps, participants were asked to write down the assumed purpose of the experiment. Also, they were invited to take part in a follow-up of the experiment 1 week later. All participants were debriefed when the experiment was finished.

### Data analysis

#### Eye movement analysis

**Figure 3:**
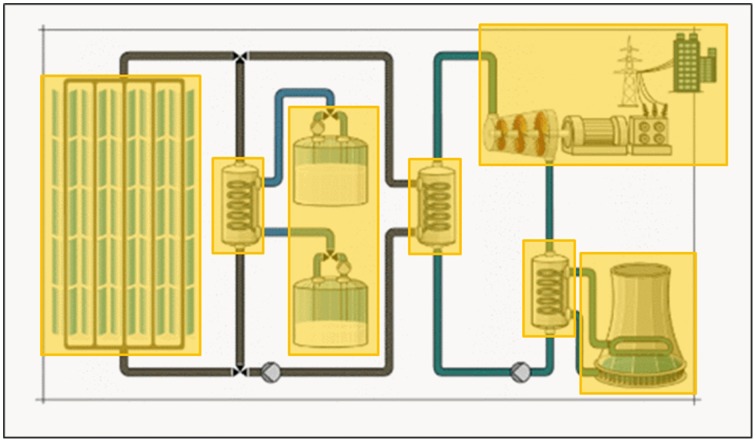
**Screenshots of the learning environment with areas of interest (yellow rectangles) used to investigate dwell time on semantic, content-relevant structures**.

We applied different eye movement parameters to investigate the specific expectations on eye movement behavior. The learning environment was accompanied by a narration, therefore we used dynamic AOIs which were temporally activated for as long as a specific region of the learning environment was concurrent with the narration. Figure 3 gives examples of the semantic AOIs applied. Note that not all AOIs were activated at the same time.

We used duration of dwell**—**defined as the sum of durations for all gaze data samples that hit an area**—**to analyze how long participants dwelled on an AOI. AOIs in the learning content contained learning relevant information and were used to determine the mean of durations of dwell on these AOIs. Examples of AOIs are the turbine, the cooling tower, single pipes, and salt tank. The dwell times on all AOIs were summed up for the final analysis.

**Figure 4:**
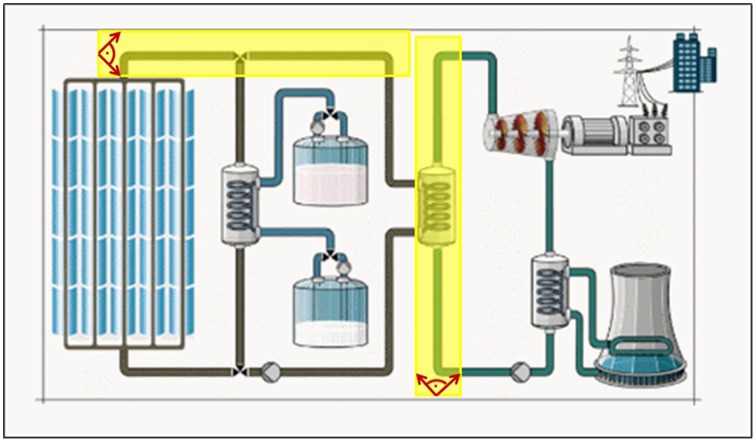
**Representations of two exemplary AOIs (yellow) which were applied to analyse saccades corresponding to the learning content and pre-training in direction**.

To find out how the pre-training affected saccades that were performed during the learning phase we used defined AOIs corresponding to description in the narration and the pre-training. For example, Figure 4 demonstrates two yellow AOIs on pipes filled with fluids running from left to right and upwards, respectively. Vectors within a 90° angle between every two fixations that hit the AOIs were recorded and aggregated to represent the number of saccades accomplished. We counted only vectors with the correct direction corresponding to the direction flow of the fluids and, thus, eye movements initiated in the pre-training. This procedure was repeated for all areas in the learning content that required direction interpretation.

To answer questions about group differences on coherence of strings we computed the Levenshtein distance for each participant within the respective groups (Levenshtein, 1966). The Levenshtein distance is the minimum of insertions, deletions, and substitutions to transform one string of fixations into another string. All fixations on AOIs of one participant were arranged in a chronological sequence, for example, B-A-B-C-B-C-B-A (each character represents a fixation on an AOI). Then, we compressed the strings meaning that repeated fixations on the same area were collapsed into a single one, for example B-A-A-B-B-B-C was transferred into a string with four characters, namely B-A-B-C (Holmqvist, 2011). First, we calculated pairwise distances between each participant and its other group members in a matrix. Because each group comprised many strings (one string for each participant), we calculated the mean of all distances from one participant to each of his/her group members divided by n-1. A low Levenshtein distance means that few operations are necessary to transfer one string into another string. This occurs when both strings are rather similar. High Levenshtein distances, on the contrary, represent dissimilar strings suggesting that many transformations are necessary to bring all strings down to a common string.

All participants had normal or corrected-to-normal vision. With respect to the obtained gaze data, the tracking ratio for the learning environment was at 92 % and the mean deviation for both eyes was at 0.69° indicating a good quality of eye movement data (Holmqvist, 2011).

#### Coding of drawings

One week after the experiment, participants were asked to create a drawing of the solar plant they learned about the week before. They were provided colored pencils and were instructed to draw a picture of the system including arrows to indicate the direction of fluid flow. The drawing product was coded as follows: one point was assigned per each component that was represented in the solar plant, one point was assigned for correct placement of a component within the system, and one point for correct labeling of a component. In total, there were nine components and therefore a maximum score of 27 could be achieved. In addition, the number of arrows which represented the direction of flow was counted and the quality of the cycles was evaluated. The latter was realized by assigning one point for a correct oil-cycle, one point for a correct salt cycle, and one point for a correct water cycle. An incomplete cycle or open cycles received 0.5 points. Here, a maximum score of 3 could be achieved. Taken together, the quality of the drawings was described by three measures: component quality (max. 27 points), number of arrows, and cycle quality (max. 3 points).

## Results

### Pre-analyses

For all statistical tests, we used an alpha level of 0.05. Table 1 displays the descriptive statistics (means and standard deviations) for all reported variables. Whenever reporting significant *t*-tests we report Cohen's d as effect size. Here, *d* ≤ 0.20 is a small effect size, *d* = 0.50 is a moderate effect size, and *d* ≥ 0.80 is a large effect size (Cohen, 1992). Whenever we report a significant analysis of variance (ANOVA) we specify the effect size by η^2^. An effect size η^2^ = 0.01 is small, η^2^ = 0.06 is moderate, and η^2^ ≥ 0.14 is considered large.

**Table 1:** **Means (and standard deviations) for all variables of interest in total and by condition (because of ANCOVAs adjusted means are reported for learning outcomes)**.

		**Conditions**		
	**Total (*n* = 44)**	**No-Training (*n* = 22)**	**Pre-Training (*n* = 22)**	***p***
Prior knowledge, %	5.83 (3.60)	5.61 (3.15)	6.06 (4.07)	0.681
Grade in physics (1–6)	2.35 (1.05)	2.23 (1.15)	2.48 (0.96)	0.440
**COMPREHENSION MEASURES**				
Learning outcomes, %	54.80 (14.47)	49.75 (14.52)	59.86 (14.52)	0.027
Structures, %	74.17 (16.43)	72.07 (16.49)	76.27 (16.49)	0.404
Processes, %	47.12 (19.29)	39.17 (19.36)	55.07 (19.36)	0.010
Functions, %	37.27 (15.48)	32.14 (15.53)	42.41 (15.53)	0.035
**SELF-REPORT MEASURES**				
Mental effort (1–9)	4.91 (2.03)	4.82 (2.32)	5.00 (1.75)	0.771
Cognitive load (1–9)	3.89 (2.10)	4.05 (2.40)	3.73 (1.80)	0.622
Stress (1–9)	3.82 (2.18)	3.86 (2.25)	3.77 (2.16)	0.892
**EYE MOVEMENT DATA**				
Dwell time, total (s)	99.05 (26.02)	102.37 (26.46)	95.74 (25.85)	0.370
Saccades, total	24.62 (13.53)	24.18 (12.55)	25.07 (14.77)	0.843
Coherence, total	25.07 (5.20)	24.94 (18.94)	25.20 (5.77)	0.863

First, we tested effects of prior knowledge on learning outcomes to determine whether prior knowledge should be considered in further tests of our hypotheses on learning outcomes. Within both conditions learning outcomes were highly correlated with the last physics grade (no-training: *r* = −0.765, *p* < 0.001; pre-training: *r* = −0.561, *p* = 0.007): better grades were associated with higher learning performance as assessed by the domain specific posttest (coding of grades: 1 = *very good*; 6 = *fail*). Moreover, groups did not differ on last grade in physics, *t*_(42)_ = −0.780, *p* = 0.440. For this reason, we included last physics grade as a covariate to investigate whether pre-training had effects on learning outcomes (analysis of covariance). There were also no group differences with respect to the prior knowledge test, *t*_(42)_ = −0.414, *p* = 0.681. However, pretest and learning outcomes were not correlated for either condition (no-training: *r* = 0.385, *p* = 0.077; pre-training: *r* = 0.230, *p* = 0.303). Therefore, pretest score was not included as covariate.

### Learning outcomes

When analyzing the overall learning outcomes, we found a significant pre-training effect, *F*_(1, 41)_ = 5.291, *p* = 0.027, η^2^_*p*_ = 0.114. In line with our expectations, the pre-training group learned more about the presented content as compared with the no-training group. Next, we examined the subscales of the posttest and tested whether this effect holds for knowledge on structures, processes, and functions. There was a non-significant effect of pre-training on knowledge about structures, *F*_(1, 41)_ = 0.710, *p* = 0.404, η^2^_*p*_ = 0.017. However, there was a significant and large effect on processes, *F*_(1, 41)_ = 7.372, *p* = 0.010, η^2^_*p*_ = 0.152, as well as on functions, *F*_(1, 41)_ = 4.779, *p* = 0.035, η^2^_*p*_ = 0.104, indicating more effective learning in favor of the pre-training group. In sum, the pre-training group showed better learning outcomes as measured by the domain-specific post-test. This superiority is in particular due to knowledge about the processes and functions of the to-be-learned technical system.

Finally, we also examined the drawings participants in the pre-training group were required to reproduce the movements of the circle cue. This procedure was part of a manipulation check to monitor whether participants in the experimental group consciously perceived the pre-training. In total, we received 17 drawings, 14 of which specified the correct direction by arrows and 16 indicated more than one cycle, but only three drawings had a close resemblance to the schematic illustration of the pre-training as demonstrated in Figure 2.

### Self-report measures on experiences during learning

The group condition had no effect on experienced cognitive load, *t*_(42)_ = 0.497, *p* = 0.622. There was a highly negative correlation between cognitive load and the learning outcomes, *r* = −0.783, *p* < 0.001, which was also found within both conditions (pre-training: *r* = −0.663, *p* = 0.001; no pre-training: *r* = −0.865, *p* < 0.001). The less cognitive load participants experienced during the learning phase the better their learning outcomes were.

Concerning mental effort we found no effect of condition, *t*_(39)_ = −0.0294, *p* = 0.771. Overall, mental effort was negatively associated with learning outcomes, *r* = −0.508; *p* < 0.001. The less mental effort had to be invested to process the learning environment the better learning outcomes were. This trend was apparent in both conditions, namely the control group, *r* = −0.587, *p* = 0.004, and the pre-training group, *r* = −0.434, *p* = 0.044.

**Figure 5:**
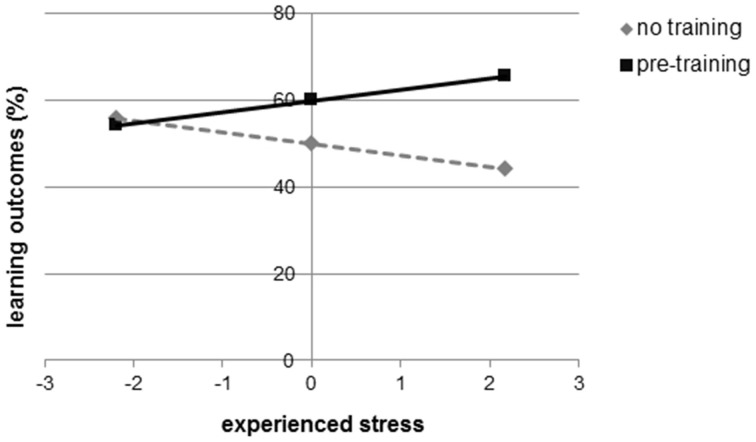
**Moderation between condition and experienced stress**.

There was no difference between both groups on stress, *t*_(42)_ = 0.137, *p* = 0.892. Although there was no correlation between reported stress and the learning outcomes in the pre-training group, *r* = 0.202, *p* = 0.368, there was a correlation between reported stress and learning outcomes in the no-training group, *r* = −0.566, *p* = 0.006. In order to test whether the relations between stress and learning outcomes differed significantly between conditions, we tested for an interaction effect using a regression in which learning outcomes were predicted from condition, experienced stress (moderator), and the interaction between condition and stress. Again, we controlled for grade in physics (Hayes, 2012). The predictors were transformed using grand mean centering. There was a significant effect of condition, *b* = 0.503, *SE* = 0.222, *t* = 2.268, *p* = 0.029, and no effect of stress, *b* = −0.004, *SE* = 0.060, *t* = −0.059, *p* = 0.953. The interaction effect, however, was significant, *b* = 0.268, *SE* = 0.101, *t* = 2.665, *p* = 0.011. We followed up this finding with simple slopes analysis where we investigated the relationship between condition and learning outcomes at low, moderate, and high levels of experienced stress (Figure 5). When participants experienced low stress levels, there was a non-significant relationship between condition and learning outcomes, *b* = −0.081, *t* = −0.217, *p* = 0.829. When stress levels were moderate, we found a significant relationship between condition and learning outcomes, *b* = 0.503, *t* = 2.268, *p* = 0.029, indicating the pre-training group performed better. At high levels of experienced stress, we found a highly significant relationship, *b* = 1.087, *t* = 4.550, *p* < 0.001, showing that the learning performance of the no-training group dropped when participants experienced higher levels of stress. In sum, the positive effects of the pre-training condition become more evident the more stress learners experience; the pre-training has no effect when learners experience low stress.

### Eye movement behavior

First, as part of our manipulation check, we tested whether the pre-training group followed the black circle as intended by the pre-training procedure. Therefore, we measured for how long the participants in the pre-training group dwelled on the black circle cue during the pre-training (Figure 2). On average, participants demonstrated cue obedience and focused on the circle for 81.45% (*SD* = 0.96) of the time.

Our first hypothesis on eye movement behavior stated that the pre-training group should dwell longer on the relevant learning contents. There was no significant effect of pre-training, *U* = 149.00, z = −0.92, *p* = 0.370. However, there was a positive correlation between duration of dwell on relevant areas and learning outcomes, *r* = 0.380, *p* = 0.019.

Next, we analyzed direction-conform saccades that were performed during the learning phase. There was no overall effect of pre-training on saccades, *t*_(36)_ = −0.200, *p* = 0.843. In addition, there was no correlation between number of performed saccades and learning outcomes, *r* = 0.075, *p* = 0.653. Finally, testing the hypothesis whether the eye movements would be more coherent in the pre-training group we found no effect of training, *U* = 174.00, *z* = −0.190, *p* = 0.863.

### Delayed posttest

All participants were invited to a follow-up posttest 1 week later. In total, 34 participants took part in the follow-up test. Data sets from two participants were excluded from the follow-up analyses as they underwent the test on more or less than seven days after the main experiment, leaving 16 participants in each condition.

We tested the effect of pre-training on three quality measures: correctness of components, cycle correctness, and number of arrows. There were no significant effects, all *p*s > 0.05. Next, we checked for correlations between learning outcomes in the main experiment and the quality of drawing in the delayed posttest when controlling for physics grade (Table 2).

**Table 2:** **Overall correlations between learning outcomes at the first test time and the quality of drawings produced at the second test time when controlled for last grade in physics**.

	**Overall**	**Control**	**Pre-training**
Component correctness	0.423^*^	0.364	0.355
Cycle correctness	0.466^**^	0.499	0.556^*^
Arrow number	0.471^**^	0.415	0.657^**^

* p < 0.05;

** p < 0.01.

Remarkably, overall learning outcomes correlated with all quality measures. When analysing these findings per condition, only two positive correlations in the pre-training group remained. The correctness of the cycles and the number of arrows implemented to represent direction flow correlated with learning outcomes. Both drawing quality measures are associated with knowledge about processes.

## Discussion

Based on recent approaches to model eye movements and the pre-training principle, the present study investigated the effects of an eye movement pre-training to foster comprehension of a technical system. We assumed that such pre-training would also reflect on oculomotor aspects in terms of eye movement parameters. In line with our expectations, the pre-training facilitated knowledge acquisition of the technical system. However, it did not influence learners' gaze behavior in the expected way. In addition, we found a relationship between experienced cognitive load, mental effort, experienced stress, and learning outcomes, indicating that the more load, effort, and stress learners experienced the poorer their learning performance was. However, this effect could not be ascribed to the pre-training of eye movements. We found that the effect of pre-training on learning performance changes as a function of experienced stress: the pre-training “assisted” learners who experienced moderate or high levels of stress. In sum, we found a clear positive effect on learning outcomes, some mixed effects with respect to self-report measures, and unexpected non-significant results on eye movement behavior. Last, a delayed posttest requiring participants to draw a picture of the learning material showed no effect of condition, but revealed associations between the quality of the picture and the learning outcomes at the first testing time which could only be ascribed to the pre-training group. The delayed posttest has an explorative character and can be considered a starting point for further research when it comes to visualizations of motion knowledge and long-term effects.

Training gaze had a positive effect on learning outcomes and successfully fostered the comprehension of a technical system. This was especially true for knowledge about processes and functions which can be considered more elaborated knowledge as compared to knowledge about structures (Kriz and Hegarty, 2007; Kalyuga and Hanham, 2011). One might argue that the performance scores were rather in the middle range leaving space for improvement. Note, however, that the learning environment was presented only once to learners who had hardly any prior knowledge of the learning contents. In addition, the posttest was extensive and exhausted all aspects of the learning environment. It is suggested that novice learners can benefit from repeated exposures of particularly complex learning environments (Lowe and Boucheix, 2011). To test the effectiveness of our pre-training we restricted the presentation time to only one viewing round.

The findings on experienced stress show that effective interventions can alleviate negative consequences of stress and preserve performance at a good level. In line with Schwabe et al. (2010), we confer that experienced stress could shed some light on the outcomes of multimedia effects and could help explaining multimedia effects in greater detail. More sophisticated measurements of stress might help investigate different facets of experienced emotional states such as challenge or hindrance in encoding or retrieval (Pekrun and Linnenbrink-Garcia, 2014).

In general, we found no effects of training on gaze behavior as assessed by dynamic and semantic areas of interest. There was just one correlation showing that the longer participants looked on areas which were temporally focused by the narration the better their learning outcomes were. However, an effect of pre-training was not found. In our analyses, we accumulated eye movement information over all temporal AOIs so that dwell time, number of saccades, and similarity were represented by one single score, respectively. This is rather a coarse-grained approach and does not allow for insights into the development of eye movement behavior during the course of learning. More fine-grained analyses might depict the development of eye movements and, thus, cognitive processing over time in more detail. Vector-based analyses are very promising in this respect and should be made applicable to complex materials (Jarodzka et al., 2010b). Similar to findings by Thomas and Lleras (2007), we can conclude that the intervention was successful although differences in eye movements were not “carried over when participants were free to look” (p. 668) during inspection of the visual representation.

Our pre-training was successful and can be regarded as a supplement to the pre-training principle described by Mayer (2009). However, it is important to make a differentiation. Instructing students to learn about components of a system requires conscious processes. Moreover, such instructions tell the students that they should memorize the names of the components because there will be subsequent information on the causal relationships between these components. In contrast, delivering an eye-movement pre-training without revealing the learning content and the specific connection to the learning material might have worked in a different way as no content processing was incorporated. To examine this conjecture, we asked participants to write down their assumptions about the purpose of the experiment. Only one participant detected the actual link between the eye movement pre-training and the learning content. Most participants perceived the pre-training to be part of the calibration process of the eye tracking device. Therefore, we have reason to believe that our pre-training worked unconsciously similar to the aforementioned findings on problem solving (Grant and Spivey, 2003; Thomas and Lleras, 2007). On the other hand, when explicitly asked to sketch the pre-training, most participants in the pre-training group correctly remembered the cue and its motions. We cannot tell in how far embodied movements of the pre-training phase triggered the mental animation during the learning process. Memory traces of the motions might have been built without the necessity to perform them in the further course. To test our tentative explanations, it is sensible to collect think aloud data during learning in order to gain some insight into the learners' internal processes and maybe even detect mental animation processes which could not be captured by our eye movement parameters. Alternatively, the pre-training might have boosted the participants' concentration without affecting their eye movement behavior or the comprehension of the motions in the learning environment. However, to rule out alternative interpretations diverse variations of eye movement pre-trainings have to be tested, for instance, pre-training eye movements incongruent to the learning environment or just a random sequence of eye movements. Such control conditions could be contrasted with trainings focusing on the static aspects of the learning content, for example the components of the solar plant, to test in how far this would elicit comprehension about structures. A significant correlation between dwell time on the structures of the material and learning outcomes, especially knowledge about structures, *r* = 0.497, *p* = 0.001, indicates that this might be the case. Moreover, the findings of this experiment could be used to investigate whether pre-training would be also beneficial for other learning materials, such as dynamic representations or non-technical contents.

Finally, some methodological concerns should be mentioned: due to the pre-training intervention the pre-training condition was longer. Both groups received identical learning material, with the exception that the pre-training group additionally received an eye movement pre-training. One might raise the objection that the no-training group should have received some other treatment to keep the overall time constant. Then, however, we would have tested two different trainings not knowing how participants would react in a control group without any support. There is evidence that keeping the overall learning time constant by reversing the order of pre-training and learning phase does not *per se* promote learning (Experiment 3 in Mayer et al., 2002). However, as this study on this type of eye movement pre-training is the first of its kind, further studies testing different materials, and target groups in different (experimental) settings should follow.

So far, the framework applied has effectively discriminated poor learners from good learners in terms of learning outcomes. Results were reported on an overall level and on the level of the subscales. Despite good reliabilities, the subscales were intercorrelated (all *ps* < 0.001). This problem was not addressed by previous research using this framework (Hmelo-Silver and Pfeffer, 2004; Kalyuga and Hanham, 2011) and should therefore receive more attention. This is a drawback that might make the functions-processes-structures framework less attractive. On the other hand, it should be discussed whether the subscales reflecting knowledge of structures, processes, and functions must be orthogonal. As proposed by Kalyuga and Hanham (also: Kalyuga et al., 2010), there is a hierarchical interdependence between these knowledge constructs, either from general to specific or specific to general knowledge. The latter approach reflects the fact that knowledge about structures is necessary to understand processes and functions.

Overall, we present a new approach to pre-training by using eye movements to foster comprehension and mental animation in a static picture. The pre-training was successful in at least two ways: (a) it supported participants' understanding of the processes and functions of a technical system, and (b) it revealed that the negative relationship between experienced stress and learning outcomes can be compensated by a non-verbal eye movement pre-training.

### Conflict of interest statement

The authors declare that the research was conducted in the absence of any commercial or financial relationships that could be construed as a potential conflict of interest.

## Appendix

### English excerpt of the narration which accompanied the static representation of the solar plant

The first cycle is called oil cycle. This cycle consists of a solar field and pipes filled with thermal oil. The second cycle is called water-steam cycle. The pipes of this cycle are filled with water and steam, respectively. This cycle consists of a turbine, a generator, a cooling tower and two warm exchange heaters. The third and last cycle is called salt cycle, because its pipes transport melted salt. In the following, the single cycles and its components will be examined in more detail.

A solar field consists of several, gutter typed parabolic reflectors, which are also called solar panels. The parabolic mirrors adjust their orientation according to the position of the sun to enable a constant and high energy yield. With the first sun rays and in the course of the day the parabolic mirrors concentrate the incoming sunlight. In the focal point of the reflector there runs thermal oil in the tube. The focused sunlight increases the temperature of the thermal oil to some 400°C. As the parabolic reflectors use a wide spectral range the solar field can increase the temperature of the thermal oil even during cloudiness.

The hot thermal oil runs to a heat exchanger. The oil runs through the heat exchanger and gives off heat to water circulating in the adjacent cycle. The heated thermal oil runs again through the tube in the solar field where its temperature can be increased again. By heating the steam in the heat exchanger the temperature and consequently the specific volume increase. The so called live stream leaves the heat exchanger at high pressure. The steam runs through pipes and flows through moving and stationary blades of a turbine. In general, the turbine operates similar to many consecutive windmills. This way the steam's temperature and pressure decrease. A generator is connected to the turbine. The generator transforms kinetic energy into electric energy. The electricity supplies single households via a network. When the cooled steam leaves the turbine it condenses into water in the cooling tower. The water runs back to the heat exchanger where it vaporizes all over again.
